# Tracking Aromatic Amines from Sources to Surface Waters

**DOI:** 10.1021/acs.estlett.4c00032

**Published:** 2024-04-10

**Authors:** Özge Edebali, Simona Krupčíková, Anna Goellner, Branislav Vrana, Melis Muz, Lisa Melymuk

**Affiliations:** † RECETOX, Masaryk University, Faculty of Science, Kotlářská 2, 611 37 Brno, Czechia; ‡ UFZ Helmholtz Centre for Environmental Research, Department of Effect Directed Analysis, Permoserstr. 15, 04318 Leipzig, Germany

**Keywords:** aromatic amines, mutagenicity, wastewater, azo dyes, sampling, indoor air

## Abstract

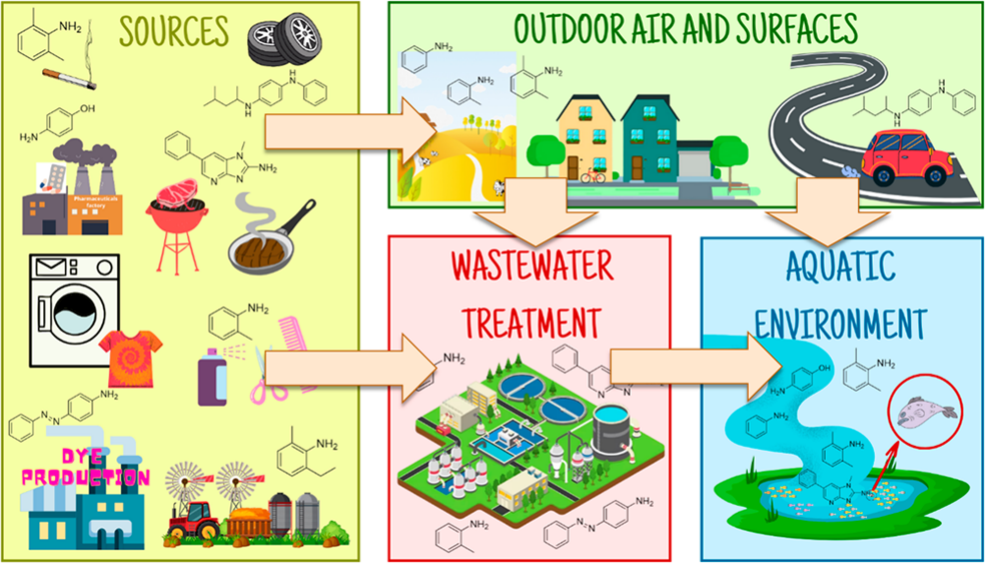

This review examines the environmental occurrence and fate of aromatic amines (AAs), a group of environmental contaminants with possible carcinogenic and mutagenic effects. AAs are known to be partially responsible for the genotoxic traits of industrial wastewater (WW), and AA antioxidants are acutely toxic to some aquatic organisms. Still, there are gaps in the available data on sources, occurrence, transport, and fate in domestic WW and indoor environments, which complicate the prevention of adverse effects in aquatic ecosystems. We review key domestic sources of these compounds, including cigarette smoke and grilled protein-rich foods, and their presence indoors and in aquatic matrices. This provides a basis to evaluate the importance of nonindustrial sources to the overall environmental burden of AAs. Appropriate sampling techniques for AAs are described, including copper-phthalocyanine trisulfonate materials, XAD resins in solid-phase extraction, and solid-phase microextraction methods, which can offer insights into AA sources, transport, and fate. Further discussion is provided on potential progress in the research of AAs and their behavior in an aim to support the development of a more comprehensive understanding of their effects and potential environmental risks.

## Introduction

Aromatic amines (AAs) are a group of compounds with environmental and industrial importance, including numerous compounds identified as mutagenic and/or carcinogenic.^1−3^ They can be found in wastewater (WW) because of their use in the production of dyes, pesticides, polymers, and pharmaceuticals, as well as a range of other poorly characterized sources.^3−6^ More than one in eight of all identified or suspected human carcinogens are either AAs or substances with the potential to transform into an AA, which emphasizes these as a significant group of human carcinogens.^7^ Epidemiological investigations have provided compelling evidence linking AAs to a risk of bladder cancer with a particularly heightened susceptibility observed among smokers.^8−10^ Major rivers, many of which are important drinking water sources, have been found to exhibit significant genotoxic/mutagenic activity,^11−16^ and bioassays have identified AAs as key contributors to the observed genotoxic and mutagenic effects.^4^ While a focus for much work on AAs has been industrial WW, broader efforts are needed to enhance our understanding of environmental contamination by mutagenic AAs from multiple matrices—from the indoor environment through to outdoor air, surfaces, WW, and surface water. Recent work targeting specific AA tire additives, notably *N*-(1,3-dimethylbutyl)-*N*′-phenyl-1,4-benzenediamine (6PPD),^17−19^ has highlighted the importance of AA emissions to aquatic systems from product use. A comprehensive evaluation of AAs necessitates tools and sampling strategies to support the source identification of AAs and probable pathways of entry to the environment. This review evaluates sources of AAs and the link between nonindustrial anthropogenic emissions and contamination of WW and surface water and evaluates the current sampling techniques suitable for AA monitoring to provide a basis for strategies to better evaluate the impact of AAs in the environment.

## Structure and Properties

AAs contain one or more amino groups attached to an aromatic ring. The compound class is diverse and ranges from aniline to highly complex structures with conjugated aromatic or heterocyclic structures and various substituents. On the basis of the number of substituents on the nitrogen atom, AAs are classified as primary, secondary, tertiary, and quaternary amines (Table 1, Table S1). They are relatively polar and possess a strong ability to interact via hydrogen bonds.^20^ The *N*-substituents can be aliphatic, aromatic, or mixed. Amines behave as bases thanks to the free electron pair on the nitrogen atom of the amino group, except for quaternary ammonium compounds. Their basicity increases with an increasing electron density at the nitrogen atom. The chemical stability of AAs is impacted by the interaction between the free electron pair of the amino nitrogen and the delocalized π-orbital system of the adjacent aromatic ring(s). This interaction decreases basicity, as do electron acceptor substituents on the aromatic ring (e.g., −Cl, −NO_2_). Alternatively, electron donor substituents, such as −CH_3_ or −OR, present in *meta*- and *para*-positions increase basicity; however, those in *ortho*-positions of the aromtic ring can sterically impede the amino group’s protonation and decrease the basicity. Such behavior is well illustrated by the acidity constant p*K*_a_ of protonated aniline and toluidines.^20^

**Table 1:**
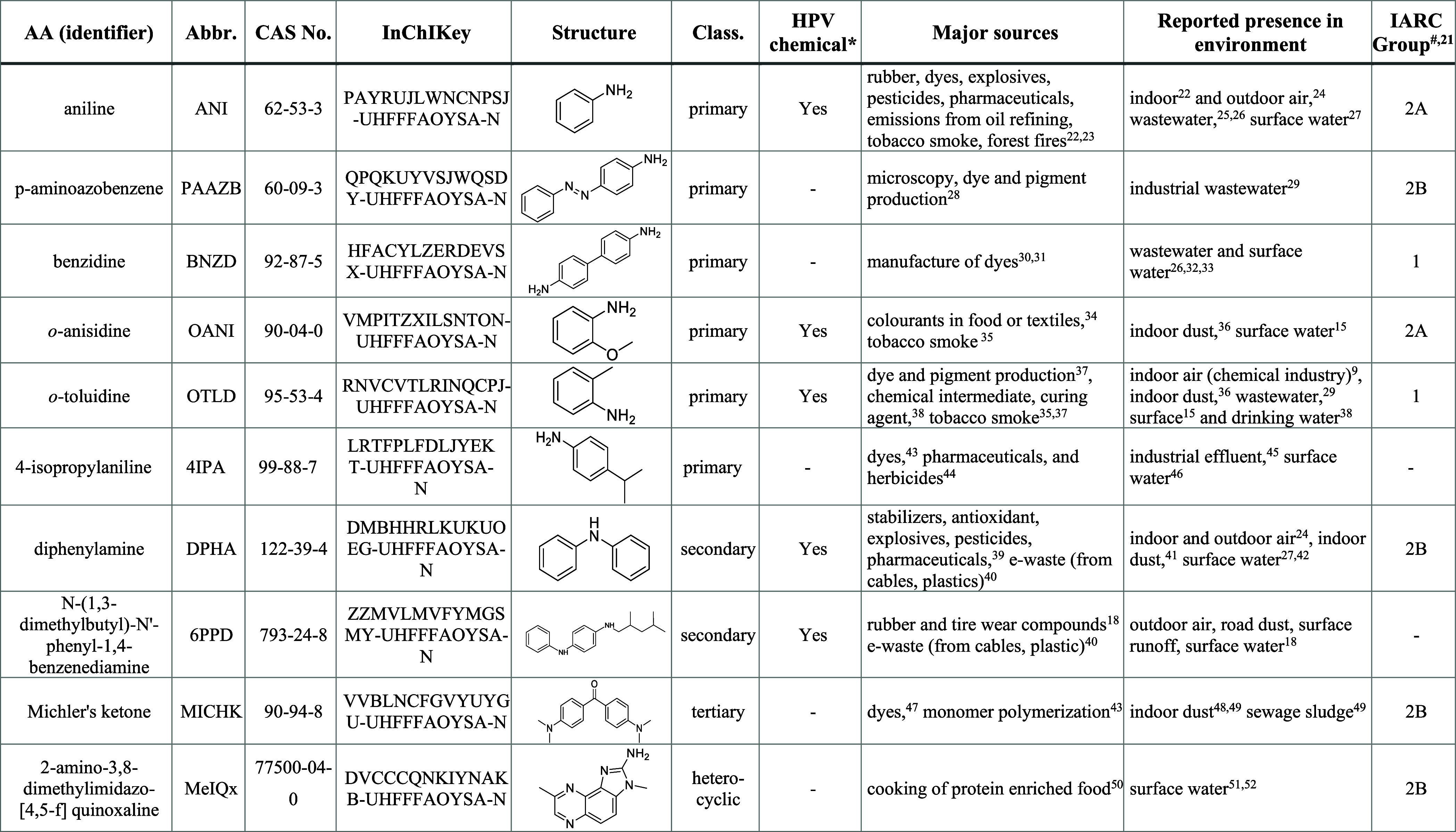
Structures, Properties, Major Sources, and Hazard Indicators of the Selected AAs^21−52^ An extended list of AAs with their physical–chemical properties is found in the Supporting Information Table S1.

*HPV according to US EPA; HPV indicates “high production volume” chemical (>500 t/y). ^#^International Agency for Research on Cancer (IARC): Group 1, carcinogenic to humans; Group 2A, probably carcinogenic to humans; Group 2B, possibly carcinogenic to humans. −, not classifiable as carcinogenic.

## Potential Sources

**Figure 1:**
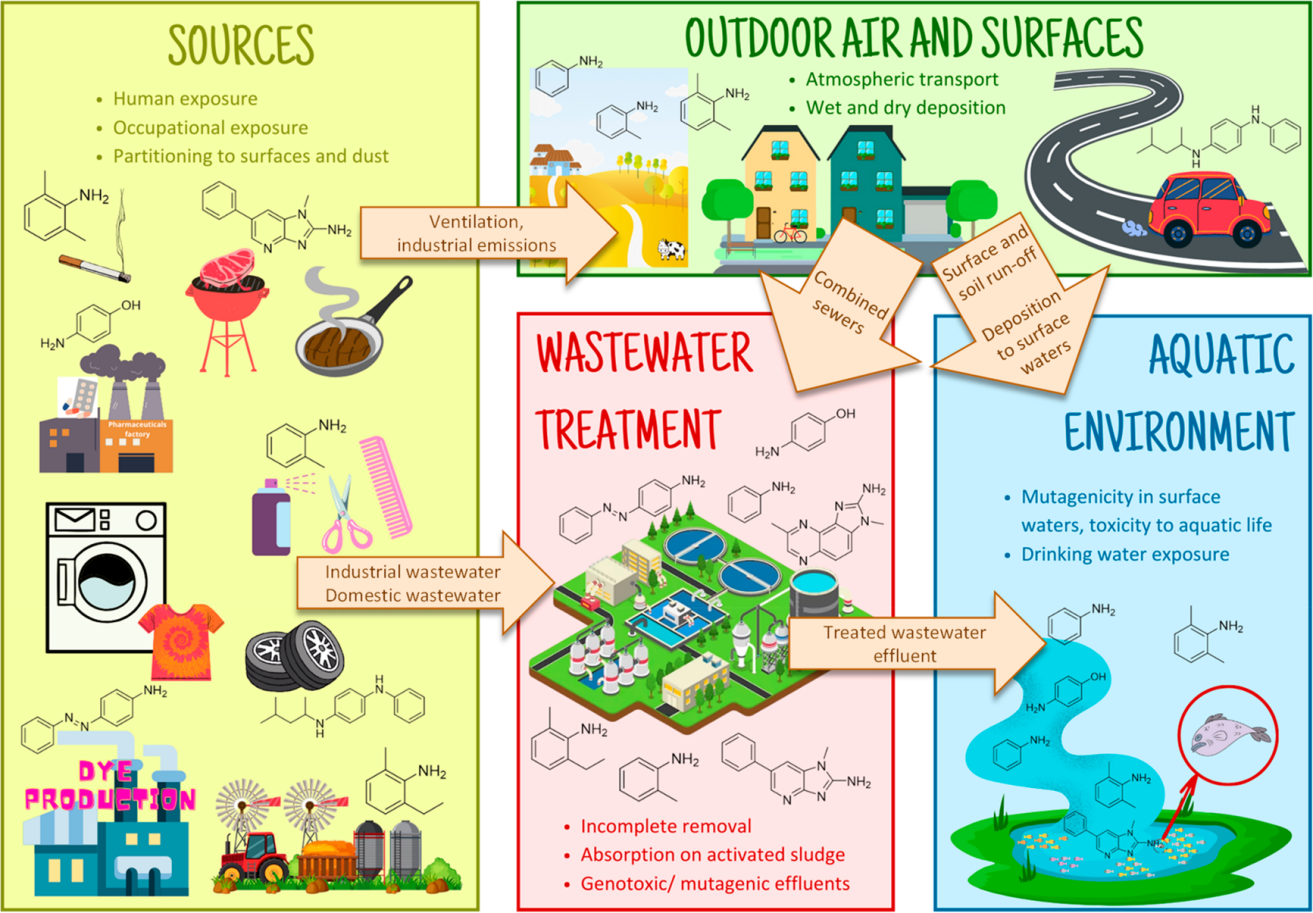
Examples of potential sources of AAs originating from both indoor environments and industrial sources contribute to WW pollution. The AAs present in insufficiently treated WW contribute to the burden of mutagenicity in surface waters. The structures depicted correspond to possible sources.

The sources listed in Table 1 can be grouped as indoor sources, which contribute initially to indoor air and dust and are subsequently transferred to the outdoor environment and industrial sources. Major industrial AA sources are the production of dyes, pharmaceuticals, pesticides, and tire rubber.^3^ Both indoor and industrial sources can impact WW treatment plants (WWTP) and eventually contaminate water bodies; however, the link between AAs from indoor environments to surface waters via WWTP has not yet been investigated. Figure 1 provides an overview of the potential sources and important pathways through which AAs can enter and affect the environment. Four main sources of AAs receive the most attention: azo dyes, smoking, grilled protein-rich foods, and rubber. However, numerous other processes can form AAs and may have substantial relevance to local scales.

### Azo Dyes

One of the most significant industrial uses, and by extension, sources of AAs, is the production of azo dyes.^53^ Azo dyes are widely used in coloring of textiles and papers, which comprise up to 70% of all dyes,^54^ and can transform into AAs under various conditions, including exposure to sunlight, heat, acidic or alkaline conditions, and enzymatic or bacterial reaction.^25,55,56^ The textile industry has high water consumption and produces complex effluent; fabric dyeing and treatment are estimated to be responsible for 20% of global WW.^57^ The amount of treatment applied to this effluent varies; up to 50% of the azo dyes produced annually enter the environment through direct discharge or losses during the dyeing process and textile washing.^56^ Conventional WW treatment methods involving light exposure, chemical treatments, or activated sludge cannot fully degrade azo dyes because of their stability and xenobiotic nature.^58^ One approach for the removal of azo dyes from WW uses microbial biocatalysis, which, under anaerobic conditions, may reduce the electrophilic azo group in the dye molecule to produce AAs.^54,59,60^ The presence of AAs in some surface waters has been specifically linked to textile dyeing facilities.^61^

Beyond textile WW treatment, several other transformation processes can generate AAs from azo dyes. Platzek et al.^62^ identified that skin bacteria can transform azo dyes from textiles to AAs on the skin surface. Weber and Adams^63^ and Macguire^64^ identified anaerobic transformation of azo dyes to AAs in sediments, and in general, azo dyes are susceptible to environmental transformation to AAs under a range of conditions.^65^

Additionally, AAs are found as impurities in materials colored by azo dyes, including primary AAs in colored paper napkins^66^ and clothing,^67−71^ with Brüschweiler et al.^68^ finding 19 of 153 clothing samples with individual AAs at levels >30 mg/g.

### Hair Dyes

AAs, such as toluidines, *p*-phenylenediamines, toluene diamines, and aminophenols, are commonly used as primary intermediates and binders in the formulation of commercial oxidative (permanent) hair dyes.^72^ AAs have been reported in hair dyes, henna, and dyed hair samples with reported concentrations of 14–109 mg/g in hair dyes where the highest levels were in darker color dyes and the lowest were in natural hennas.^73^ Hair dyes and dyed hair samples were dominated by 1,3- and 1,4-phenylenediamine with concentrations of these individual compounds in the mg/g range.^73^ The presence of AAs in hair dyes has been flagged as a potential health concern given their potential as skin sensitizers, mutagens, and carcinogens,^74^ and some epidemiological evidence has linked exposure to AAs via hair dyes with bladder cancer.^75^

### Rubber and Tires

Another source of AAs is the production and use of rubber and plastic materials. AAs are commonly used as accelerators and antioxidants in rubber and plastic products^76^ and can be 5–10% of the weight of a car tire,^77^ with *p*-phenylenediamine derivatives most commonly used.^78^ Occupational hazards associated with rubber production have long been recognized, and mutagenic activity has been identified in the raw chemicals and ambient rubber dust and fumes.^79^ However, recent concern has focused on environmental hazards: a transformation product of 6PPD, 6PPD-quinone, has been linked with acute mortality events in salmon populations caused by stormwater runoff.^80,81^ 6PPD is an AA used as one of the major additive antioxidants in tires;^17^ however, beyond 6PPD, multiple other *p*-phenylenediamine derivatives are commonly used in rubber products, including *N,N*′-bis(1,4-dimethylpentyl)-*p*-phenylenediamine (77PD), *N*-phenyl-*N*′-cyclohexyl-*p*-phenylenediamine (CPPD), *N,N*′-diphenyl-*p*-phenylenediamine (DPPD), *N,N*′-di-2-naphthyl-*p*-phenylenediamine (DNPD), and *N*-isopropyl-*N*′-phenyl-1,4-phenylenediamine (IPPD).^78^ Abrasive wear, particularly from tires, and subsequent mobilization to air or runoff to surface water, is taken to be the main environmental release pathway,^78^ and these tire-associated AAs have been detected in surface waters^82−88^ and recently in urban air.^89,90^ Given the growing concern regarding tire rubber-derived chemicals and their transformation products, these compounds have received attention in recent dedicated reviews.^17,18,91,92^

### Tobacco Smoke

Tobacco smoke contains a complex mixture of chemicals, including AAs, which are formed during the combustion of tobacco leaves.^35,93−95^ Tobacco smoking is a known source of human exposure to AAs,^96,97^ and a significant correlation has been found between concentrations of AAs and nicotine in indoor dust,^36^ as well as elevated levels of AAs in indoor spaces where smoking takes place.^22,24,98^ Harmane and norharmane have been detected in tobacco smoke.^99^

### Foods

AAs are also found in some food products^100^ because food components, such as nitrites and nitrates, can react with amino acids to form AAs. Heterocyclic aromatic amines (HAAs) are generated during cooking, especially in grilled or fried protein-rich foods.^101,102^ HAAs are commonly detected in heated animal-derived foods because of the high content of creatine, which is needed for their generation, and have been reported in different types of cooked meat,^103,104^ including smoked and baked sausage,^105^ chicken,^106,107^ pork,^108^ and beef patties.^109^ Total levels of HAAs are typically in the 10–100 ng/g range.^103^

The specific compounds often reported in foods are 2-amino-1-methyl-6-phenylimidazo[4,5-*b*]pyridine (PhIP), 2-amino-3,8-dimethylimidazo-[4,5-*f*]quinoxaline (MeIQx), and β-carboline alkaloids, such as harmane, harmine, and harmalol.^100^ Levels vary by type of meat, cooking methods, and ingredients used.^101,103,105,106,108,109^ Concentrations of PhIP, MeIQx, harmane, and norharmane increase with longer frying times and higher temperatures.^103^ In addition, the presence of AAs in the coloring of food packaging can be a further contributor to their presence in food.^110^ AAs resulting from dietary exposure can be identified in urine.^97^

### Other Sources

AAs are widely used in many other industrial processes, including the synthesis of pesticides, pharmaceuticals, and explosives.^23,72^ They are also used in the production of epoxy resins,^72^ polyurethane,^72^ cosmetics, and food.^111^ Consequently, there is a wide range of products found to have AA content, including kitchen utensils,^112^ food contact materials,^113^ herbicide formulations,^114^ and cosmetics.^111^

AAs are also metabolites of nitroarenes. For example, 1-nitropyrene, one of the major polycyclic aromatic compounds emitted in diesel exhaust,^115^ is metabolized to 3-aminobenzanthrone and 1-aminopyrene. These AAs were found at elevated levels in the urine of workers exposed to diesel combustion.^116^ AAs can also be formed in the environment via the transformation of other synthetic organic compounds, such as reduction or hydrolysis of isocyanates and pesticides. For instance, the herbicides alachlor and metolachlor can degrade into 2,6-diethylaniline and 2-ethyl-6-methylaniline,^117^ the pesticide naptalam degrades into 1-naphthylamine,^118^ 3,4-dichloroaniline can be released from herbicides diuron and propanil,^119^ and 2-amino-*N*-isopropylbenzamide can be released from the herbicide betazone.^120^

## Hazards and Regulation

AAs are both carcinogenic and mutagenic. AAs are frameshift mutagens and require metabolic activation to exert mutagenicity.^1,3,121^ They can undergo metabolic activation in the human body (*N*-acetylation, oxidation, and conjugation with glucuronic acid); the metabolism varies by specific compound.^122,123^ The metabolic activation is mediated by cytochrome P450 enzymes and leads to the formation of *N*-hydroxy-AAs through an initial oxidation step, which is an electrophile that can form DNA adducts. The second step involves the esterification of *N*-hydroxy-AA by sulpho- or acyltransferase, which results in the formation of the final nitrenium ion, (R_2_N:^+^) that reacts with the DNA of affected organisms.^124^

A common pathway for detoxification of HAAs (such as IQ, MeIQx, PhIP, AαC, etc.) in humans is glucuronidation.^123,125^ This has also been shown for 4-aminobiphenyl, 2-naphthylamine, and 1-naphthylamine.^126^ Metabolites are then excreted in urine.^122,125,126^

Exposure to AAs has been linked to a variety of health effects, including bladder cancer, liver cancer, and kidney damage.^9,127^ The link between AA exposure and cancer in humans was discovered in 1895 through increases in bladder cancer in workers in the dye industry.^128^ In the 1930s, the working group of Wilhelm C. Hueper postulated that 2-naphthylamine can trigger the growth of bladder tumors, which led to a ban on its production and handling in many countries.^128^ Phenylenediamine exposure has been linked to the development of lung and skin cancers.^94^ HAAs are carcinogenic and raise the risk of several malignancies, including colorectal cancer.^129^ For several HAAs, the range of specific mutagenicity for mammalian cell lines employing the Hprt gene or Ef-2 gene as reporters is comparable with that for *Salmonella typhimurium*.^100^ Despite clear hazard information for a subset of AAs, many have limited information on toxicity. Novel techniques incorporating computational toxicology^130,131^ or proteome profiling^132^ have improved available information for azo dyes and some AAs, and there is clear potential for the further application of such techniques for less studied AAs, such as the metabolites of brominated azo dyes.^132^

The carcinogenic and mutagenic properties of AAs have led to widespread regulations on their use. In the European Union, 22 AAs are classified as carcinogens and some are also classified as mutagens by CLP Regulation (EC) No. 1272/2008.^133^ IARC and the World Health Organization (WHO) have classified five AAs, namely benzidine, 2-naphthylamine, *o*-toluidine, 4-aminobiphenyl, and 4,4′-methylenebis(2-chloroaniline), as known human carcinogens (Group 1), while others are classified as probable or possible carcinogens (Groups 2A and 2B).^21^ They are also listed in Appendix 8 of REACH regulation EC No. 1907/2006 as restricted substances.^134^ Several AAs, including diethylmethyl benzenediamine and melamine, have been added to the European Chemicals Agency’s endocrine disruptor (ED) assessment list.^135^

## Environmental Sampling Techniques

Sample preparation and analysis techniques for AAs have been previously reviewed, including sample preparation^2,136^ and detection/quantification techniques.^54,136^ Herein, we focus on sampling techniques with specific relevance to AAs, particularly novel in situ sampling. Several materials have been employed in environmental sampling to specifically enrich AAs/HAAs from different matrices. One of the most used materials is a blue pigment: copper phthalocyanine trisulfonate (CPT). The discovery of CPT as an efficient ligand to trap polycyclic compounds is based on the inhibitory effect of hemin on mutagenic activities of polycyclic aromatic hydrocarbons^137^ and of the carcinogenic heterocyclic amine Trp P 1.^138^ Hemin inhibits the mutagenicity of compounds via the porphyrin structure forming complexes with the planar surface of mutagens.^139^ This interaction led to the development of samplers where CPT bonds covalently to supporting polymers, such as chitin, cotton, and rayon. Because of the blue color of CPT, the samplers are named blue cotton, blue rayon, and blue chitin, respectively. The adsorbent has a planar structure and, therefore, a high affinity for aromatic rings and planar polycyclic compounds.^140^ The compounds adsorbed can be easily eluted by shaking with a methanol/ammonia solution, usually with a ratio of 50:1 (v/v). The ammonia likely aids in the dissociation of the complex by binding to the central metal ion within the ligand.^141^

Early studies applied these samplers to mutagenic HAAs in complex matrices. Hayatsu et al.^140^ used blue cotton to extract mutagenic HAAs from urine, river water and cooked beef extracts. Blue cotton was also used to monitor mutagenic activity in seawater^142^ and to enrich HAAs from dialysis fluids,^143^ human plasma,^144^ and human cataractous lenses.^145^ Yamashita et al.^146^ applied blue cotton to cigarette condensates and detected two yet unmeasured HAAs in cigarette smoke.

An advancement to blue cotton was made by using rayon as the supporting material to create a “blue rayon” (BR). It contains at least double the amount of blue pigment compared with blue cotton and can be used in situ but also be packed in columns like blue cotton.^141^ Sakamoto and Hayatsu^147^ deployed BR directly in river water. The BR extracts exerted a strong mutagenic effect, and the novel AA mutagens phenylbenzotriazoles (PBTA) were identified as the cause of the high mutagenicity in the river.^148−151^ Novel environmental AA mutagens 2,3- and 2,8-phenazinediamine were identified with a combination of sampling with BR and Ames test in a tributary of the Elbe River.^4^ BR extracts from the Elbe River showed an increase in mutagenic response with metabolic activation, thereby suggesting the contribution of AAs to the mutagenicity of the river.^152^

Blue chitin is produced as a powder and, therefore, can be used as a solid-phase extraction (SPE) sorbent.^153^ It contains 2–4 times more pigment, is more selective to compounds that contain three or more aromatic rings, and yields higher recoveries than BR but, most importantly, has a much higher reproducibility.^153,154^ Although more quantitative results can be obtained by employing blue chitin, it has not yet been developed for use as an on-site extraction material. One challenge is that all three materials are weakly mutagenic themselves, and if they are not properly cleaned before deployment, they can cause blank mutagenicity.^155^

To sample an even wider range of AAs, including monoaromatic amines, XAD resins have been used in an SPE column to extract AAs from liquid samples. Kummrow et al.^155^ compared the mutagenicity of BR and XAD extracts of river water receiving dye plant discharges and observed higher mutagenicity in XAD extracts, thereby indicating that polycyclic compounds found in the BR extracts do not account for all of the observed mutagenicity.^61^ In a comparison between XAD resin columns, BR, and blue chitin with river water samples, the XAD extracts showed lower mutagenicity than the blue chitin extracts, while BR extracts showed higher mutagenicity than blue cotton extracts, which may be because CPT is a more efficient material than XAD for adsorption of polycyclic mutagens.^154^

Headspace solid-phase microextraction (SPME) using polyethylene glycol–graphene oxide sol–gel coating has been used with water samples.^156^ As expected for headspace techniques, the more volatile primary AAs could be quantified, but because the polymer has a delocalized π-electron system, it is also promising for compounds with multiple aromatic rings as strong π–π stacking interactions can be formed.

To detect airborne AAs, applications with different materials have been reported in studies. Zhang et al.^90^ used active air samplers with quartz fiber filters to collect fine particulate matter (PM2.5) in Chinese cities and successfully quantified tire-wear-related *p*-phenylenediamine antioxidants in the extracts. Passive air sampling using polyurethane foam disk air samplers has also been successful in the detection of airborne chemicals associated with tire wear, including phenylenediamines.^89^ XAD resins have been used for many decades to study HAAs in air, including MeIQx and 2-amino-3,4,8-trimethylimidazo[4,5-*f*]quinoxaline (DiMeIQx) in cooking fumes from frying meat.^157^ XAD resins processed into an active needle trap device by the sol–gel method have also been used for air sampling of AAs.^158^

It should be mentioned that none of the summarized methods is entirely specific to sample AAs and also enriches other organic substances; for blue cotton/rayon/chitin, it is mainly other polycyclic substances, but for XAD, even aliphatic compounds can be enriched.^61,153^ This provides the opportunity for the integration of monitoring of AAs into broader environmental monitoring programs.

## Environmental Presence and Fate

### Indoor Environments

As with many industrial- and consumer-product-related chemicals, indoor levels of AAs are expected to be higher than outdoor levels, although there are limited data to support this. In Italy, indoor air concentrations of aniline were higher than outdoor levels, which ranged from 10 to 1700 ng/m^3^.^159^ Elevated AA levels are well-documented in occupational indoor environments, e.g., in rubber and dye industries;^9,79^ therefore, we focus on nonindustrial indoor environments.

Only a few studies address AA levels in nonindustrial indoor air (Table S2) and dust (Table S3), and direct comparison among existing studies is difficult because of limited overlap in investigated AAs. A clear distinction is shown between the levels in smoking and nonsmoking environments. Higher levels of AAs have been detected in smoking environments compared with nonsmoking environments. In Canada, aniline levels were found to be 34 ± 19 ng/m^3^ in smoking environments and 11 ± 9 ng/m^3^ in nonsmoking households,^22^ while a Turkish study found aniline levels ranging from 6–21 ng/m^3^ in smoking-impacted indoor air compared with 1–4 ng/m^3^ in nonsmoking areas.^24^ In China, a study found that the concentrations of certain AAs in indoor dust were significantly associated with nicotine levels, thereby suggesting tobacco smoke as a source of AAs indoors.^160^

Beyond this distinction between smoking and nonsmoking indoor spaces, few conclusions can be drawn about indoor levels of AAs and their dominant sources. In restaurant kitchens, the total concentration range of 2,4,5-trimethylaniline, 2-naphthylamine, and 4-aminobiphenyl in indoor air samples was between 34 and 6090 ng/m^3^.^161^ The presence of AAs in indoor dust has only been investigated in a few studies (Table S2), with AA levels typically in the ng/g range.^36^ Recent studies have identified the presence of a number of AA antioxidants, including 6PPD,^41^ in indoor dust.^162,163^

### Urban Air and Road Dust

Until recently, aniline was the most commonly quantified AA in outdoor air, and higher levels of primary AAs were related to urban and industrial areas (Table S4).^159^ In the past few years, the focus of outdoor urban air and dust samples has shifted to the AA antioxidants with studies reporting levels of 6PPD and other *p*-phenylenediamine derivatives in urban^89,90^ and highway-adjacent air^164^ and particularly in urban surface dust (e.g., road and parking lot dust at up to μg/g levels)^165^ (Table S5).

### Wastewater Effluents

While WW treatment techniques are effective at removing some AAs, many AAs are not effectively removed during WW treatment,^166^ or can be formed from parent compounds (e.g., azo dyes) under anaerobic conditions during WW treatment.^167^ As a result, WW effluents are typically considered one of the most important inputs of AAs to surface waters. Effluents of WWTPs receiving industrial, domestic, and hospital WW are reported to be genotoxic and/or mutagenic and dominated by frameshift mutagens.^168−171^ There is clear evidence of AA occurrence in industrial WW (Table S6).^4,26,33,172^ Muz et al.^4^ identified diaminophenazines as compounds responsible for a large portion of mutagenicity in WW effluents from an industrial area in Germany. Particular AA profiles can be characteristic of specific industrial discharges: *o*-toluidine and 3,4-dichloroaniline were identified as characteristic compounds occurring in blast furnace and steel rolling processes, 4-dichloroaniline is additionally in dye WW, and *p*-chloroaniline and 3,5-dichlorobenzeneamine are in WWs from printing and dyeing plants.^173^

Municipal WW often comes from combined sewer systems, and under wet weather conditions, a substantial load will originate from impervious surface runoff. Tire- and road-wear-derived AAs have been identified in WW influent and effluent attributed to road runoff collected by combined sewer systems,^85,87,174^ and the detection of these compounds in urban rivers during dry weather further suggests WW inputs.^84^

Domestic WW, which contains a mix of WW from toilets, kitchen, bath, laundry, floor, and surface cleaning has been shown to be as genotoxic as WW that contains both industrial and domestic WW, thereby exhibiting elevated mutagenicity with metabolic activation.^175^ However, uncertainty remains regarding the compounds responsible for these mutagenic effects. Human metabolites of xenobiotics, such as glucoronide conjugates, can often be found in municipal WWs.^176−178^ During WW treatment, metabolites may be released from their parent compounds because of their bacterial activity. For AAs, this pathway was not been investigated. To our knowledge, no studies report the occurrence or levels of AAs in domestic WW. However, the presence of both banned azo dyes and AAs in textiles,^71^ personal care products,^73^ and indoor dust^36^ suggest the potential for AA presence in domestic WW from cleaning and laundering.^175^ Future research should address this knowledge gap and explore whether there is a causative relationship between the occurrence of AAs in the indoor environment and the mutagenicity observed in domestic WWs.

### Surface Water, Sediments, and Groundwater

Because of the presence of an amino group in their structure, AAs exhibit relatively high polarity and moderate to high water solubility.^2^ This characteristic suggests that they are likely to be found primarily in the hydrosphere if released into the environment. This enables easy permeation through soil and a high possibility of groundwater contamination,^179,180^ which poses a potential risk to drinking water sources.^181^ Levels of selected AAs can further increase from surface/groundwater levels over the course of chlorination-based drinking water treatment; Jurado-Sánchez et al. observed a 10-fold increase in AAs within a drinking water treatment plant,^181^ which suggests that the environmental contamination in drinking water source waters can be further increased during drinking water treatment.

Numerous studies have reported the presence of AAs in rivers (Table S7). The first report of AAs in surface waters came in the 1970s with the detection of 3,4-dichloroaniline and 2-chloroaniline in the Rhine River.^182^ Bioanalytical findings have indicated the contribution of AAs to the mutagenic effects observed in surface water samples across the globe, including North America,^183^ Europe,^4,14,184−187^ Brazil,^61^ and Japan.^187,188^ Despite these findings, chemical/quantification data on AAs in surface waters are limited: primary AAs have been quantified in Turkish river and seawater;^27^ Italian,^189^ German,^15^ and Iranian rivers;^190^ and Chinese surface waters.^85,86,191,192^ However, most of these studies are limited to method validation, and extensive screenings have not been done. There is growing literature covering tire rubber-associated AAs in surface waters^17,18^ given the particular concern about their aquatic toxicity,^80^ and increases in surface water concentrations of tire rubber-associated AAs are documented during rain events, which suggests a clear pathway from surface runoff to surface waters.^82^

Outside of surface water, quantitative information about AAs is even more limited. *o*-Toluidine, *p*-chloroaniline, 2,4-dichloroaniline, 2,5-dichloroaniline, 3,4-dichloroaniline, and 3,5-dichloroaniline have been detected in groundwater in an industrially polluted area north of Milan, Italy,^193^ while Urbaniak et al.^194^ ubiquitously detected AAs in sediment from surface water bodies in the USA, especially aniline, *o*-anisidine, and 4-chloroaniline. Sediments from a WW lagoon contained the highest concentrations of AAs, thereby suggesting that sewage discharges are major sources of these chemicals in the aquatic environment. The risk quotients calculated for the chemicals in sediments suggested a potential threat to aquatic organisms.

Weakly mutagenic AAs can exhibit elevated mutagenicity because of synergistic interactions with carboline alkaloids and other nitrogen-containing compounds present in surface waters.^184^ This complexity makes it more challenging to pinpoint the mutagenicity drivers in surface waters, and understanding the sources of AAs is crucial to unraveling the complex compound mixtures and their respective adverse effects.

### Environmental Fate

The most important elimination pathways of AAs in surface waters include microbial transformation, sorption to suspended solids, and sediment and covalent association with dissolved humics.^20^ The efficiency of removal depends on several factors, including the structural features and concentration of AAs, temperature, pH, and redox conditions. In addition, the presence of suitable cosubstrates may play an important role in biotransformation.^20^

Biological degradation of AAs is also a relevant elimination pathway in WW treatment processes under aerobic or anaerobic conditions.^20^ Oxidative biotransformation of primary AAs results in the replacement of an amino substituent by a hydroxy functional group: the aromatic ring can be oxidized by dioxygenases and dehydrogenases to form pyrocatechols, and those subsequently undergo an ortho-cleavage of the ring to easily degradable nonaromatic products.^195,196^ In general, aromatic ring substituents with chloro, sulpho, or nitro groups decrease the degradation rate, whereas carboxy or hydroxy substituents increase it.^197,198^ AAs can also polymerize under aerobic conditions.^20^ Compounds with tertiary or quaternary nitrogen are biologically more stable.^20^

AA interactions with solid phases consist of cation exchange and hydrophobic partitioning.^20,199^ The speciation and sorption of AAs in the environment are highly pH-dependent processes.^20,199^ AAs can associate with humic substances via ionic interactions, hydrophilic partitioning, and covalent binding (concretely nucleophilic addition of the amino group to electrophilic moieties of humic molecules and oxidative mechanisms).^20,200,201^ The latter is less important in surface waters because they typically contain low concentrations of dissolved organic matter.^20,201^

In comparison with biodegradation, abiotic oxidation by dissolved oxygen and photochemical degradation by sunlight are less important removal pathways of AAs.^20,202^ However, the transformation of 6PPD to the toxic 6PPD-quinone occurs under abiotic oxidative conditions and is documented to occur in the environment.^203^ Most amines are resistant to hydrolysis in aqueous solutions.^20^

Only some AAs are persistent in the aquatic environment. Phenylurea pesticide-derived anilines, e.g., 4-chloroaniline and 3,4-dichloroaniline, are considered persistent toxic AAs.^204,205^ This has been confirmed by Zhou et al.,^166^ who showed that 2,6-dimethyl aniline, 2-chloro-4-nitroaniline, 2,6-diethylaniline, and 3,4-dichloroaniline were not removed during aerobic sewage treatment simulation tests, which indicates these AAs will enter surface water and pose a potential risk to aquatic organisms.

## Future Perspectives

Numerous studies have highlighted sources of AAs; however, the precise mechanisms by which they are transported from industrial or indoor origins into the environment remain poorly understood. Data on AAs in indoor air, dust, and WW systems are scarce. Although existing studies have touched upon indoor dust and air concentrations, they have predominantly focused on primary amines or only on aniline. Moreover, the specific contribution of domestic WW to the overall presence of AAs in municipal sewage, especially compared with the contributions from industrial sources and road runoff, has not been assessed. An essential area for future exploration lies in evaluating the contribution of nonindustrial sources to the presence of AAs in WW and surface waters and examining the causal link between the presence of AAs and the observed mutagenicity in both WW and surface waters.

## Supporting Information Available

Extended list of AAs and their properties and representative levels of AAs in indoor air, indoor dust, outdoor air, outdoor dust, wastewater, and surface water (PDF)
