# Supplementary material for: Tracking Aromatic Amines from Sources to Surface Waters
Source: Environ Sci Technol Lett. 2024 Apr 10;11(5):397–409. doi: 10.1021/acs.estlett.4c00032 (PMC11097632; doi:10.1021/acs.estlett.4c00032)
Supplement: Supplementary file 1 — ez4c00032_si_001.pdf [file ez4c00032_si_001.pdf]

Tracking aromatic amines from sources to surface waters

Özge Edebali[1,+] Simona Krupčíková[1,+] Anna Goellner[2], Branislav Vrana[1], Melis Muz[2], Lisa Melymuk[1,*]

[1] RECETOX, Masaryk University, Faculty of Science, Kotlářská 2, 611 37 Brno, Czechia

2 Department Effect-Directed Analysis, Helmholtz Centre for Environmental Research - UFZ, Permoserstrasse 15, 04318 Leipzig, Germany

[+]Özge Edebali and Simona Krupčíková contributed equally to the study and have shared first authorship.

* Corresponding author: Lisa Melymuk, lisa.melymuk@recetox.muni.cz

Contents

Table S1 - Properties of selected AAs S2
Table S2 - Levels of AAs in indoor air S9
Table S3 - Levels of AAs in indoor dust S9
Table S4 - Levels of AAs in outdoor air S11
Table S5 - Levels of AAs in outdoor dust S12
Table S6 - Levels of AAs in wastewater S12
Table S7 - Levels of AAs in surface water S14

Table S1 - Properties of selected AAs; all structures were made with ChemDraw 19.1 and values of octanol-water partition coefficient (K_{ow}), boiling point (t_B), soil adsorption coefficient (K_{oc}) – were generated using the US Environmental Protection Agency's EPI Suite^{TM} EPIWEB 4.1, pK_a values are from PubChem [1]

Name	Abbreviation	CAS No.	Structure	M* g mol^{-1}	Classification	pK_a at	log K_{oa}	log K_{ow}	log K_{oc}	t_B (°C)
1-naphthylamine	1NAPA	134-32-7	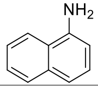	143.19	1°	3.9	7.593	2.25	3.48	293.14
2,2-dimethylbenzidine		84-67-3	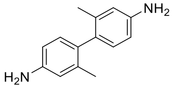	212.30	1°		11.610	3.02	3.87	393.08
2,4,5-trimethylaniline		137-17-7	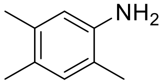	135.21	1°		6.264	2.72	2.30	241.78
2,4,6-trichloroaniline		634-93-5	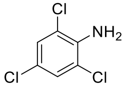	196.46	1°		8.019	3.01	3.65	273.12
2,4-diaminotoluene		95-80-7	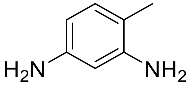	122.17	1°		7.657	0.16	2.08	270.43
2,4-xylidine		95-68-1	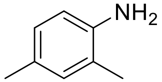	121.18	1°		5.703	2.17	2.08	223.42
2,5-diaminotoluene		95-70-5	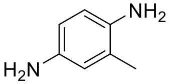	122.17	1°		7.677	0.16	2.08	270.43
2,5-dichloroaniline		95-82-9	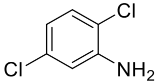	162.01	1°		7.117	2.37	2.08	245.76
2,6-diethylaniline		579-66-8	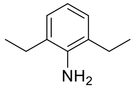	149.24	1°			3.15	2.84	259.25
2,6-xylidine	26XYL	87-62-7	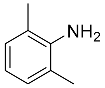	121.18	1°	3.9	5.827	2.17	2.09	223.42
2-amino-1-methyl-6-phenylimidazo[4,5-b]pyridine	PhIP	105650-23-5	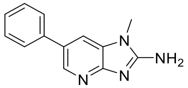	224.27	1°, HAA		13.174	2.16	4.05	452.10

2-amino-3,8-dimethylimidazo-[4,5-f]quinoxaline	MeIQx	77500-04-0	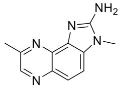	213.24	1°, HAA	2.2	12.192	1.09	2.77	441.80
2-amino-3-methylimidazo[4,5-f]quinoline	IQ	76180-96-6	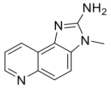	198.23	1°, HAA	8.48	12.301	1.57	3.53	424.77
2-amino-4-nitroanisol		99-59-2	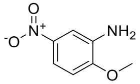	168.15	1°		9.211	1.55	1.57	303.88
2-amino-4-nitrophenol		99-57-0	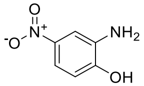	154.13	1°		11.755	0.99	1.93	317.38
2-amino-4-nitrotoluene	2A4NT	99-55-8	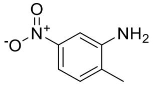	152.15	1°	2.3	15.269	-0.76	2.56	455.55
2-amino-5-chloro-*p*-toluenesulfonic acid		88-53-9	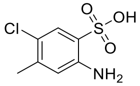	221.66	1°		9.637	-0.89	1.00	393.55
2-amino-5-nitrophenol		121-88-0	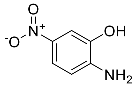	154.13	1°		11.485	0.99	1.93	317.38
2-amino-5-nitrothiazol		121-66-4	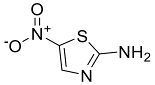	145.14	1°, HAA		10.491	0.64	1.45	287.80
2-amino-6-methoxybenzothiazol		1747-60-0	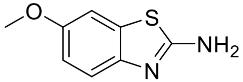	180.23	1°, HAA		11.575	2.08	3.06	334.37
2-amino-6-nitrobenzothiazole		6285-57-0	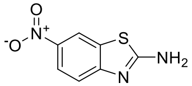	195.20	1°, HAA		12.491	1.82	3.27	366.81
2-amino-9*H*-pyrido[2,3-b]indole	AalphaC	26148-68-5	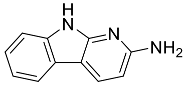	183.21	1°, HAA		13.631	2.47	3.49	366.26
2-aminobiphenyl	2AMB	90-41-5	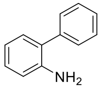	169.23	1°	3.8	8.064	2.84	3.24	326.21
2-aminopyridine	2APY	504-29-0	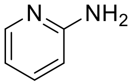	94.12	1°, HAA	6.9	7.472	0.53	1.65	193.56

2-bromoaniline	2BRA	615-36-1	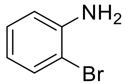	172.02	1°	2.5		1.97	2.06	235.40
2-chloroaniline		95-51-2	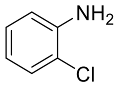	127.57	1°		5.557	1.72	1.87	216.05
2-ethyl-6-methylaniline		24549-06-2	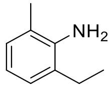	135.21	1°		6.560	2.66	2.37	241.78
2-methoxy-4-nitroaniline		97-52-9	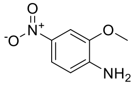	168.15	1°		9.291	1.55	1.57	303.88
2-naphthylamine	2NAPA	91-59-8	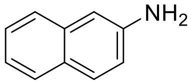	143.19	1°	4.2	7.760	2.25	3.47	293.14
2-nitroaniline	2NITA	88-74-4	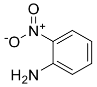	138.12	1°	-0.3	7.468	2.02	1.72	272.55
3,3’-dichlorbenzidine	33DCB	91-94-1	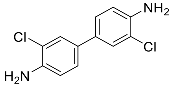	253.13	1°	2.7	12.445	3.21	3.87	407.27
3,3'-dimethylbenzidine	33DMB	119-93-7	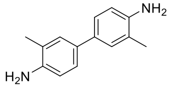	212.29	1°	4.6	10.930	3.02	3.87	393.08
3,4,5-trichloroaniline		634-91-3	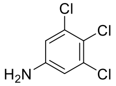	196.46	1°		7.819	3.01	2.82	273.12
3,4-dichloroaniline		95-76-1	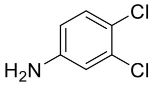	162.01	1°	2.78	5.914	2.37	2.08	245.76
3’-aminoacetophenone	3AAP	99-03-6	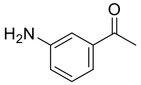	135.16	1°	3.4	7.678	0.76	1.10	258.46
3-amino-5-nitro-2,1-benzisothiazol		14346-19-1	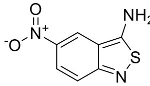	195.20	1°, HAA		10.693	1.07	3.28	366.81
3-chloroaniline		108-42-9	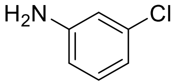	127.57	1°		6.151	1.72	2.05	216.05

3-chloro-*o*-toluidine	3CHOT	87-60-5	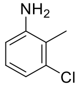	141.60	1°	2.5	6.465	2.27	2.09	234.76
4,4’-thiodianiline	44TDA	139-65-1	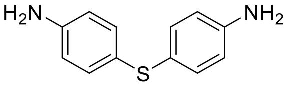	216.30	1°	4.6	11.975	2.46	3.70	389.05
4,4'-methylenebis(2-chloroaniline)	MBOCA	101-14-4	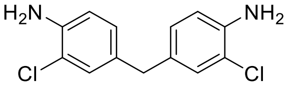	267.15	1°	3.3	12.781	3.47	4.13	404.79
4,4’-methylene-bis(2-methylaniline)	MEMDA	838-88-0	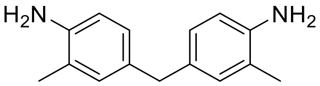	226.32	1°	5.2	11.805	3.28	4.13	390.60
4,4'-oxydianiline	44ODA	101-80-4	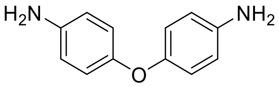	200.24	1°	4.0	10.581	2.22	3.05	367.85
4-aminobiphenyl	4AMB	92-67-1	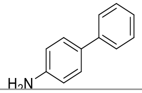	169.23	1°	4.4	8.084	2.84	3.23	326.21
4-aminophenol		123-30-8	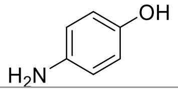	109.13	1°		7.448	0.24	1.86	241.84
4-chloraniline	4CHA	106-47-8	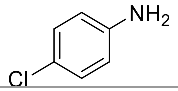	127.57	1°	4.2	6.154	1.72	1.86	216.05
4-chloro-*o*-toluidine	4CHOT	95-69-2	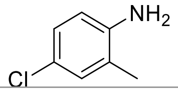	141.60	1°	3.4	6.465	2.27	2.08	234.76
4-methoxy-*m*-phenylenediamine		615-05-4	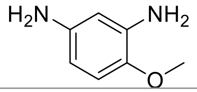	138.17	1°		8.479	-0.31	1.72	286.98
4-nitro-1,3-phenylenediamine		5131-58-8	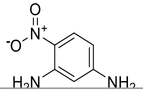	153.14	1°		16.753	-2.77	2.54	486.52
4-nitroaniline		100-01-6	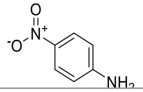	138.13	1°		8.678	1.47	1.71	272.55
4-*tert*-butylaniline		769-92-6	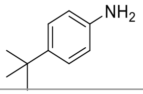	149.24	1°		6.396	2.99	2.68	244.81

5-chloro-2-methylaniline		95-79-4	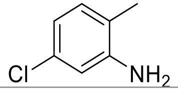	141.60	1°		6.465	2.27	2.08	234.76
aniline	ANI	62-53-3	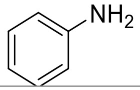	93.13	1°	4.6	4.983	1.08	1.65	183.99
azobenzene	AZOB	103-33-3	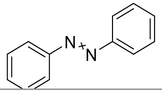	182.23	1°azo-dye	-3.0	7.078	4.11	3.29	294.03
benzidine	BNZD	92-87-5	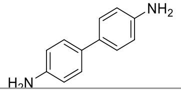	194.24	1°	4.7	10.015	1.92	3.44	369.88
4-isopropylaniline	4-IPA	99-88-7	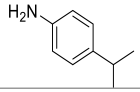	135.21	1°	5.0	6.310	2.53	2.34	230.83
dianilinomethane	DADPM	101-77-9	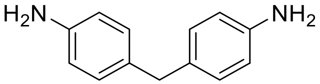	198.24	1°	5.3	10.201	2.18	3.70	367.40
m-phenylenediamine		108–45-2	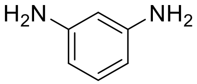	108.14	1°		8.079	-0.39	1.86	253.58
m-toluidine		108-44-1	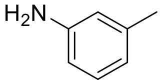	107.16	1°		5.568	1.62	2.05	204.16
o-aminoazotoluene	OAAT	97-56-3	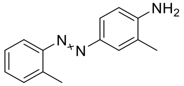	225.29	1°, azo-dye	3.0	10.176	4.29	3.16	366.43
o-anisidine	OANI	90-04-0	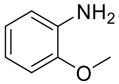	123.16	1°	4.5	6.515	1.16	1.51	224.16
o-dianisidine	ODAN	119-90-4	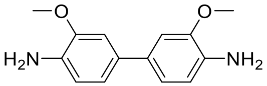	244.29	1°	4.7	12.941	2.08	3.16	417.19
o-toluidine	OTLD	95-53-4	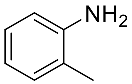	107.20	1°	4.4	5.412	1.62	1.87	204.16
p-aminoazobenzene	PAAZB	60-09-3	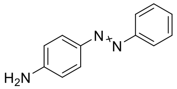	197.24	1°, azo dye	3.06	10.082	3.19	2.72	343.16

p-anisidine	PANI	104-94-9	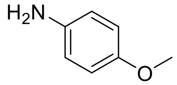	123.16	1°		6.519	1.16	1.65	224.16
p-cresidine	PCRE	120-71-8	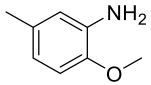	137.18	1°	4.7	7.035	1.70	1.72	242.48
p-phenylenediamine		106-50-3	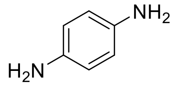	108.14	1°		7.260	-0.39	1.86	253.58
p-toluidine	PTLD	106-49-0	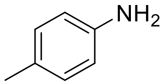	107.16	1°		5.473	1.62	2.052	204.16
p-aminodimethylaniline	DMPD	99-98-9	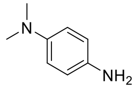	136.20	1°, 3°	6.6	7.157	1.25	2.10	241.29
anilinonaphtalene	PANA	90-30-2	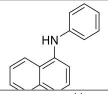	219.29	2°	4.9	9.576	4.47	4.33	363.24
N-(1,3-dimethylbutyl)-N'-phenyl-1,4-benzenediamine	6PPD	793-24-8	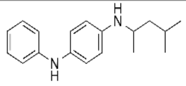	268	2°	6.73	11.542	4.68	4.84	369.67
diphenylamine	DPHA	122-39-4	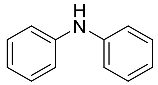	169.23	2°	0. 8	7.358	3.29	3.28	283.01
N-isopropyl-*N*'-phenyl-*p*-phenylenediamine	IPPD	101-72-4	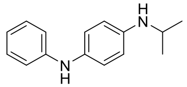	226.32	2°	6.8	10.510	3.28	4.10	341.75
Michler's ketone	MICHK	90-94-8	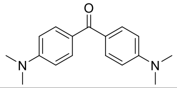	199.27	3°	2.6	11.567	3.50	3.15	373.88
N,*N*-dimethylaniline		121-69-7	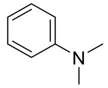	121.18	3°		4.944	2.17	1.89	169.39
quaternium-15 (free base)	Q15	4080-31-3	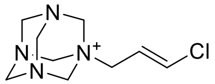	215.70	4°			-5.92	1.53	461.53
3-carboline	3CARB	244-76-8	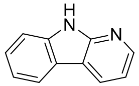	168.20	HAA	14.5	10.448	2.74	3.28	322.12

5-carboline	5CARB	244-69-9	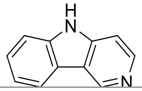	168.20	HAA	15.0	9.808	2.10	3.28	322.12
norharmane	NORH	244-63-3	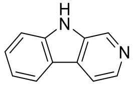	168.20	HAA	15.4	9.808	2.10	3.28	322.12
harmane	HRMA	486-84-0	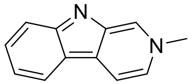	182.23	HAA	7.4	10.315	2.65	3.49	334.92
harmine	HRMI	442-51-3	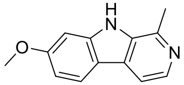	212.25	HAA	7.7	13.080	2.83	4.12	380.88

The following tables provide overviews of a selection of AAs based on major published studies covering indoor air and dust, outdoor air and surface dusts, wastewater, and surface water. We note that these are not comprehensive tables, and in particular, for the AA antioxidants/tire rubber-associated AAs identify draw attention to reviews focussing on these subsets of AAs: Jin et al.[2], Zoroufchi Benis et al.[3]and Hua et al.[4]

Table S2 – Representative levels of AAs in indoor air

AA	Levels
Aniline	187 µg m^{-3} (occupational air, USA) [5]<br>0.034±0.019 µg m^{-3} (Smoking indoor air, Canada) [6]<br>0.011±0.009 µg m^{-3} (Non-smoking indoor air, Canada) [6]<br>6.12-21.03 ng m^{-3} (Smoking indoor air, Turkey) [7]<br>1.18-4.42 ng m^{-3} (Non-Smoking indoor air, Turkey) [7]
o-toluidine	412 µg m^{-3} (occupational air, USA) [5]
Σ_3 primary AAs	0.034–6.09 ng m^{-3} (Indoor air - restaurant kitchens, China) [8]
Σ_9 primary AAs	3-207 ng m^{-3} (General indoor air, Italy) [9]
Σ_{15} primary AAs	0.7-487 ng m^{-3} (Smoking indoor air, France) [10]<br>1.1-15 ng m^{-3} (Non-smoking indoor air, France) [10]<br>1.2-442 ng m^{-3} (Smoking influence indoor air, France) [10]

Table S3 – Representative levels of AAs in indoor dust

AA	Levels
2,6-dimethylaniline (2,6-DMA)	<LOQ-117 ng/g (indoor dust, Colombia) [11]<br>6.20 - 64.5 ng/g (indoor dust, Greece) [11]<br><LOD- 45.4 ng/g (indoor dust, India) [11]<br>2.03 – 21.0 ng/g (indoor dust, Japan) [11]<br><LOD- 746 ng/g (indoor dust, Kuwait) [11]<br><LOD- 388 ng/g (indoor dust, Pakistan) [11]<br><LOD – 339 ng/g (indoor dust, Romania) [11]<br><LOD- 610 ng/g (indoor dust, Saudi Arabia) [11]<br><LOD – 905 ng/g (indoor dust, South Korea) [11]<br><LOD - 11.2 ng/g (indoor dust, Vietnam) [11]
N-phenyl-1-naphthylamine (AO-A)	4.56–120 ng/g (residential dust, China) [12]
N-phenyl-2-naphthylamine (AO-D)	3.30–170 ng/g (residential dust, China) [12]
N1-cyclohexyl-N4-phenyl-benzene-1,4-diamine (CPPD)	5.2-66.8 ng/g (indoor vehicle dust, China) [13]<br><LOQ-0.4 ng/g (house dust, e-waste region, China) [13]
DChA	< LOQ – 70.7 ng/g (residential dust, China) [12]
diAMS	< LOQ – 254 ng/g (residential dust, China) [12]<br>13.3-983 ng/g (E-waste dust, Canada)[14]<br>< MDL-25.3 ng/g (residential dust, Canada) [14]<br>< MDL-13.6 ng/g (residential dust, USA) [14]
di-n-octyl-DPA	< LOQ – 78.1 ng/g (residential dust, China) [12]
di-t-butyl-DPA	< LOQ– 10.7 ng/g (residential dust, China) [12]
DNPD	1.9-29.5 ng/g (indoor vehicle dust, China) [13]<br><LOQ-137 ng/g (house dust, ewaste region, China) [13]
DPA	8.71–129 ng/g (residential dust, China) [12]<br>81.8-439 ng/g (e-waste dust, Canada) [14]<br>< MDL-10.6 ng/g (residential dust, Canada) [14]<br>5.70-53.6 ng/g (residential dust, USA) [14]
DPPD	< LOQ – 22.2 ng/g (residential dust, China) [13]

	<LOQ-55.3 ng/g (indoor vehicle dust, China) [13]<br><LOQ-27.0 ng/g (house dust, e-waste region, China) [13]<br>nd-5.47 ng/g (Guangzhou, China)[15]<br>nd ng/g (Hanoi, Vietnam) [15]<br>nd-5.99 (Adelaide, Australia) [15]<br>nd ng/g (Illinois, U.S) [15]
IPPD	<LOQ-575 ng/g (indoor vehicle dust, China) [13]<br><LOQ-41.5 ng/g (house dust, ewaste region, China) [13]<br>2.65-37.8 ng/g (e-waste dust, Canada) [14]<br>< MDL-0.22 ng/g (residential dust, Canada) [14]<br>< MDL-1.91 ng/g (residential dust, USA) [14]
o-anisidine	<LOQ -104 ng/g (indoor dust, Colombia) [11]<br>1 - 211 ng/g (indoor dust, Greece) [11]<br><LOD- 127 ng/g (indoor dust, India) [11]<br>6.05 – 360 ng/g (indoor dust, Japan) [11]<br><LOD- 43.2 ng/g (indoor dust, Kuwait) [11]<br><LOD- 28.1 ng/g (indoor dust, Pakistan) [11]<br>2.81–111 ng/g (indoor dust, Romania) [11]<br><LOD- 156 ng/g (indoor dust, Saudi Arabia) [11]<br>6.60–146 ng/g (indoor dust, South Korea) [11]<br><LOD - 99.1 ng/g (indoor dust, Vietnam) [11]
ortho/meta-toluidine	<LOQ-9.60 ng/g (indoor dust, Colombia) [11]<br>0.89-32.1 ng/g (indoor dust, Greece) [11]<br>0.18- 11.4 ng/g (indoor dust, India) [11]<br>0.83- 18.4 ng/g (indoor dust, Japan) [11]<br><LOD- 27.8 ng/g (indoor dust, Kuwait) [11]<br><LOD- 18.8 ng/g (indoor dust, Pakistan) [11]<br>1.31- 9.30 ng/g (indoor dust, Romania) [11]<br><LOD- 19.1 ng/g (indoor dust, Saudi Arabia) [11]<br>0.76 - 73.5 ng/g (indoor dust, South Korea) [11]<br>0.34- 3.89 ng/g (indoor dust, Vietnam) [11]
p-anisidine	<LOQ -3.81 ng/g (indoor dust, Colombia) [11]<br><LOD - 2.13 ng/g (indoor dust, Greece) [11]<br><LOD- 39.5 ng/g (indoor dust, India) [11]<br><LOD – 2.18 ng/g (indoor dust, Japan) [11]<br><LOD- 9.75 ng/g (indoor dust, Kuwait) [11]<br><LOD- 4.15 ng/g (indoor dust, Pakistan) [11]<br><LOD- 5.60 ng/g (indoor dust, Romania) [11]<br><LOD - 26.7 ng/g (indoor dust, Saudi Arabia) [11]<br><LOD –3.65 ng/g (indoor dust, South Korea) [11]<br><LOD - 5.15 ng/g (indoor dust, Vietnam) [11]
p-cresidine (PCRE)	<LOQ-59.0 ng/g (indoor dust, Colombia) [11]<br><LOD-240 ng/g (indoor dust, Greece) [11]<br>0.98–143 ng/g (indoor dust, India) [11]<br>1.44 - 20.3 ng/g (indoor dust, Japan) [11]<br><LOD- 29.8 ng/g (indoor dust, Kuwait) [11]<br><LOD- 115 ng/g (indoor dust, Pakistan) [11]<br><LOD - 115 ng/g (indoor dust, Romania) [11]<br><LOD- 218 ng/g (indoor dust, Saudi Arabia) [11]<br><LOD - 87.1 ng/g (indoor dust, South Korea) [11]<br><LOD - 51.3 ng/g (indoor dust, Vietnam) [11]
77PD	<LOQ -9.6 ng/g (indoor vehicle dust, China) [13]

	<LOQ-77.6 ng/g (house dust, ewaste region, China) [13]
6PPD	< LOQ – 180 ng/g (residential dust, China) [12]<br>5.0-41.9 ng/g (indoor vehicle dust, China) [13]<br><LOQ-6.1 ng/g (house dust, ewaste region, China) [13]<br>7.31-37.7 ng/g (E-waste dust, Canada) [14]<br>< MDL-6.65 ng/g (residential dust, Canada) [14]<br>< MDL-23.7 ng/g (residential dust, USA) [14]
p-toluidine (p-TD)	<LOQ-0.56 ng/g (indoor dust, Colombia) [11]<br><LOD-11.9 ng/g (indoor dust, Greece) [11]<br><LOD- 115 ng/g (indoor dust, India) [11]<br><LOD- 5.85 ng/g (indoor dust, Japan) [11]<br><LOD- 49.4 ng/g (indoor dust, Kuwait) [11]<br><LOD- 21.4 ng/g (indoor dust, Pakistan) [11]<br>0.18- 8.90 ng/g (indoor dust, Romania) [11]<br><LOD- 21.7 ng/g (indoor dust, Saudi Arabia) [11]<br><LOD - 18.2 ng/g (indoor dust, South Korea) [11]<br><LOD - 2.58 ng/g (indoor dust, Vietnam) [11]
$\sum_{20}$AAs	0.958-42370 ng/g (Tianjin, China)[16]
$\sum_{21}$AA antioxidants	511 - 1.06×10^4 ng/g (e-waste processing facility dust, South China) [17]
$\sum_6$PPDs	ND-1747 ng/g (Tianjin, China) [16]
Michler's ketone	<MQL – 55.2 ng/g (indoor dust, Canada)[48]<br><MQL – 344 ng/g (indoor dust, China)[49]

Table S4 – Representative levels of AAs in outdoor air

AA	Levels
Aniline	50-425 ng m^{-3} (outdoor air, Italy) [9]<br>8.27±3.19 ng m^{-3} (winter mean, PM10, Turkey) [7]<br>3.29±1.06 ng m^{-3} (winter mean, gaseous air, Turkey) [7]
Benzylamine	3.55±2.01 ng m^{-3} (winter mean, PM10, Turkey) [7]<br>1.19±0.46 ng m^{-3} (winter mean, gas-phase air, Turkey) [7]
Butylamine	9.46±3.66 ng m^{-3} (winter mean, PM10, Turkey) [7]<br>2.96±1.21 ng m^{-3} (winter mean, gas-phase air, Turkey) [7]
CPPD	0.1-21 (PM2.5, urban air, China) [18]<br><LOD-0.74 pg m^{-3} (aerosols, Hong Kong) [19]<br>< MDL-0.431 ($PM_{2.5}$, China) [20]
Dibuthylamine	10.98±4.02 ng m^{-3} (winter mean, PM10, Turkey) [7]<br>4.38±2.01 ng m^{-3} (winter mean, gas-phase air, Turkey) [7]
Diethylamine	6.20±2.93 ng m^{-3} (winter mean, PM10, Turkey) [7]<br>2.88±1.63 ng m^{-3} (winter mean, gas-phase air, Turkey) [7]
Dimethylamine	6.84±3.20 ng m^{-3} (winter mean, PM10, Turkey) [7]<br>3.37±2.01 ng m^{-3} (winter mean, gas-phase air, Turkey) [7]
Diphenylamine	6.35±1.37 ng m^{-3} (winter mean, PM10, Turkey) [7]<br>1.21±0.89 ng m^{-3} (winter mean, gas-phase air, Turkey) [7]
DNPD	0.3-108 (PM2.5, urban air, China) [18]<br>< MDL-0.0356 (PM2.5, China) [20]
DPPD	0.1-13 (PM2.5, urban air, China) [18]<br><LOD-0.70 pg m^{-3} (aerosols, Hong Kong) [19]<br>0.055-2.590 ($PM_{2.5}$, China) [20]
DTPD	<LOD-2.88 pg m^{-3} (aerosols, Hong Kong) [19]
Ethylamine	6.87±3.51 ng m^{-3} (winter mean, PM10, Turkey) [7]<br>2.16±1.21 ng m^{-3} (winter mean, gas-phase air, Turkey) [7]
IPPD	0.2-104 pg m-3 (PM2.5, urban air, China) [18]<br>0.44-2.73 pg m^{-3} (aerosols, Hong Kong) [19]<br>0.00171-3.690 ($PM_{2.5}$, China) [20]
Methylamine	7.13±3.17 ng m^{-3} (winter mean, PM10, Turkey) [7]

	2.78±1.44 ng m^{-3} (winter mean, gas-phase air, Turkey) [7]
m-Toluidine	7.96±3.60 ng m^{-3} (winter mean, PM10, Turkey) [7]<br>2.15±1.45 ng m^{-3} (winter mean, gas-phase air, Turkey) [7]
N-methylaniline	7.89±3.21 ng m^{-3} (winter mean, PM10, Turkey) [7]<br>2.35±1.08 ng m^{-3} (winter mean, gas-phase air, Turkey) [7]
n-Propylamine	8.58±3.54 ng m^{-3} (winter mean, PM10, Turkey) [7]<br>2.85±1.37 ng m^{-3} (winter mean, gas-phase air, Turkey) [7]
77PD	0.1-967 (PM2.5, urban air, China) [18]<br>< MDL-2.980 ($PM_{2.5}$, China) [20]
6PPD	0.02-487 (PM2.5, urban air, China) [18]<br>0.82-6.30 pg m^{-3} (aerosols, Hong Kong) [19]<br>0.022-6.050 ($PM_{2.5}$, China) [20]
Pyrrolidine	2.55±1.61 ng m^{-3} (winter mean, PM10, Turkey) [7]<br>1.17±0.68 ng m^{-3} (winter mean, gas-phase air, Turkey) [7]
Σ_9 primary AAs	<20-100 ng m^{-3} (outdoor air, Italy) [9]

Table S5 – Representative levels of AAs in outdoor dust

**AA**	**Levels**
AO-A	<LOQ - 299 ng/g (outdoor playground dust, China) [12]
AO-D	<LOQ - 137 ng/g (outdoor playground dust, China) [12]
CPPD	< LOQ (outdoor playground dust, China) [12]<br>3.4-190 ng/g (road dust, China) [13]<br>5.8-540 ng/g (parking lot dust, China) [13]
diAMS	3.37–290 ng/g (outdoor playground dust, China) [12]
di-n-octyl-DPA	< LOQ – 4.59 × 10^3 ng/g (outdoor playground dust, China) [12]
di-t-butyl-DPA	3.22–8.07 × 10^3 ng/g (outdoor playground dust, China) [12]
DNPD	1.5-35.9 ng/g (road dust, China) [13]<br><LOQ-28.9 ng/g (parking lot dust, China) [13]
DPA	2.33–32.6 ng/g (outdoor playground dust, China) [12]
DPPD	< LOQ – 22.6 ng/g (outdoor playground dust, China) [12]<br>5.8-126 ng/g (road dust, China) [13]<br>16.4-217 ng/g (parking lot dust, China) [13]<br><LOQ-153 ng/g (road dust, Guangzhou, China) [21]<br><LOQ-697 ng/g (parking lot dust, Guangzhou, China) [21]
IPPD	<LOQ-321 ng/g (road dust, China) [13]<br><LOQ-237 ng/g (parking lot dust, China) [13]<br><LOQ-247 ng/g (road dust, Guangzhou, China) [21]<br><LOQ-307 ng/g (parking lot dust, Guangzhou, China) [21]
77PD	<LOQ-38.5 ng/g (road dust, China) [13]<br><LOQ-28.9 ng/g (parking lot dust, China) [13]
6PPD	< LOQ – 685 ng/g (outdoor playground dust, China) [12]<br>4.1-238 ng/g (road dust, China) [13]<br>13.5-429 ng/g (parking lot dust, China) [13]<br>1.5-1.9 µg/g (road tunnel dust, Germany) [22]<br>15.1-1508 ng/g (road dust, Guangzhou, China) [21]<br>11.4-5359 ng/g (parking lot dust, Guangzhou, China) [21]<br>45-1175 ng/g (road dust, Japan)[23]

Table S6 – Representative levels of AAs in wastewater

**AA**	**Levels**
1-chloro-3-nitrobenzene	120 µg L^{-1} (WWTP influent, Canada)[24]<br>0.15 µg L^{-1} (WWTP effluent, Canada) [24]
1-naphthylamine	2.4 µg L^{-1} (municipal WW influent, China) [25]
2,3-phenazinediamine	0.01−0.21 mg L^{-1} (WWTP effluent, Germany)[26]
2,4,5-trichloroaniline	388 µg L^{-1} (WWTP influent, Canada) [24]

2,4,5-trimethylaniline	68.42 ng L^{-1} (textile wastewater, Turkey)[27]
2,4-diaminoanisole	5.63 ng L^{-1} (textile wastewater, Turkey)[27]
2,4-diaminotoluene	0.0033-0.12 µg L^{-1} (WWTP, Brazil)[28]
2,4-dimethylaniline	171.92 ng L^{-1} (textile wastewater, Turkey)[27]<br>1955 µg L^{-1} (WWTP influent, Canada) [24]<br>2760 µg L^{-1} (WWTP influent, Canada) [24]<br>0.63 µg L^{-1} (Primary WWTP sludge, Canada) [24]<br>0.03 µg L^{-1} (WWTP effluent, Canada) [24]
2,4-dinitroaniline	0.27 µg L^{-1} (WWTP effluent, China) [29]
2,5-methoxymethylaniline	0.0014-0.79 µg L^{-1} (WWTP, Brazil) [28]
2,6-dimethylaniline	50.38 ng L^{-1} (textile wastewater, Turkey)[27]<br>118 µg L^{-1} (Industrial WW composite, Canada) [24]<br>121 µg L^{-1} (WW influent, Canada) [24]
2,8-phenazinediamine	0.03−0.52 mg L^{-1} (WWTP effluent, Germany) [26]
2-Chloroaniline	6.90 µg L^{-1} (Industrial water composite, Canada) [24]
2-ethoxybenzamine	0.24 µg L^{-1} (WWTP influent, Canada) [24]
2-hydroxyquinoline	0.001 7 −0.02 mg L^{-1} (WWTP effluent, Germany) [26]
2-naphthylamine	68.49 ng L^{-1} (textile wastewater, Turkey) [27]
2-nitroaniline	0.17 µg L^{-1} (WWTP effluent, China)[29]
3,3-dichlorobenzidine	2.6 – 654 µg L^{-1} (Industrial water composite, Canada) [24]<br>0.0033-0.12 µg L^{-1} (WWTP, Brazil) [28]
3,4-dichloroaniline	0.097 µg L^{-1} (WWTP effluent, China) [29]<br>483 µg L^{-1} (Primary WW sludge, Canada) [24]
3-formylindole	0.01 7 −0.05 mg L^{-1} (WWTP effluent, Germany) [26]
3-nitroaniline	<0.9 µg L^{-1} (municipal WW influent, Saudi Arabia) [30]
4- hydroxyquinoline	0.001 7 −0.01 mg L^{-1} (WWTP effluent, Germany) [26]
4,4-diaminodimethane	0.0056-0.78 µg L^{-1} (WWTP, Brazil) [28]
4,4-diaminodiphenylmethane	15.96 ng L^{-1} (textile wastewater, Turkey) [27]
4,4-oxydianiline	33.87 ng L^{-1} (textile wastewater, Turkey)[27]
4-bromoaniline	2.64 µg L^{-1} (municipal WW influent, Saudi Arabia) [30]
4-chloro-2-nitroaniline	3.5 µg L^{-1} (Industrial WW composite, Canada) [24]<br>1.02 µg L^{-1} (WWTP influent, Canada) [24]<br>0.64 µg L^{-1} (WWTP effluent, Canada) [24]
4-chloroaniline	<1.8 µg L^{-1} (municipal WW influent, Saudi Arabia)[30]<br>0.0016-0.06 µg L^{-1} (WWTP, Brazil) [28]
4-dimethyl aminopyridine	0.02−1.30 mg L^{-1} (WWTP effluent, Germany) [26]
4-ethoxybenzamine	4.57 µg L^{-1} (WWTP influent, Canada) [24]<br>0.45 µg L^{-1} (WWTP influent, Canada) [24]
4-methyl-2-nitroaniline	0.19 µg L^{-1} (WWTP influent, Canada) [24]
4-nitroaniline	0.0009-0.23 µg L^{-1} (WWTP, Brazil) [28]
6,7-dimethoxy-2-(1- piperazinyl)-4-quinazolinamine	0.001−0.35 mg L^{-1} (WWTP effluent, Germany) [26]
6PPD	1.1-59 ng/L (influent, China) [31]<br><LOQ-15 ng/L (effluent, China) [31]
aniline	4.5 ng mL^{-1} (municipal WW, Iran)[32]<br>1.1 µg L^{-1} (municipal WW influent, China) [25]<br>12.80 µg L^{-1} (Industrial water composite, Canada) [24]<br>121 µg L^{-1} (WWTP influent, Canada) [24]<br>0.05-1270 µg L^{-1} (WWTP sludge, Canada) [24]<br>0.0030-0.15 µg L^{-1} (WWTP, Brazil) [28]
benzidine	3.6 µg L^{-1} (municipal WW influent, China) [25]<br>0.37-13.4 µg L^{-1} (Industrial water composite, Canada) [24]
guanine	0.05 −0.52 mg L^{-1} (WWTP effluent, Germany) [26]
IPPD	0.63-33 ng/L (WWTP influent, China) [31]<br>0.13-28 ng/L (WWTP effluent, China) [31]
L-(+)-ergothioneine	0.01−0.39 mg L^{-1} (WWTP effluent, Germany) [26]
MBOCA	5.26 ng L^{-1} (textile wastewater, Turkey)[27]
N,*N*-dimethylaniline	0.5 ng mL^{-1} (municipal WW, Iran)[32]
o-aminoazotoluene	12.40 ng L^{-1} (textile wastewater, Turkey) [27]

o-toluidine	1.5 ng mL^{-1} (municipal WW, Iran)[32]<br>42.7 ng L^{-1} (textile wastewater, Turkey)[27]
p-aminoazobenzene	5.15 ng L^{-1} (textile wastewater, Turkey)[27]
phenylalanine	0.03−3.04 mg L^{-1} (WWTP effluent, Germany) [26]
propranolol	0.02−0.09 mg L^{-1} (WWTP effluent, Germany) [26]
telmisartan	0.09−0.65 mg L^{-1} (WWTP effluent, Germany) [26]
tryptophan	0.01−0.40 mg L^{-1} (WWTP effluent, Germany) [26]
tyrosine	0.15−3.26 mg L^{-1} (WWTP effluent, Germany) [26]
4-isopropylaniline	ppm range (Industrial WW, Brazil)[41]
Michler's ketone	<MQL–224 ng/g d.w. (WWTP sludge, China)[49]

Table S7 – Representative levels of AAs in surface water

AA	Levels
1,2-phenylendiamine	3.72 –15.68 ng L^{-1} (sea water, Black Sea) [33]
1-naphthylamine	40.03-66.12 ng L^{-1} (river water, Turkey) [33]<br>17.78 – 38.25 ng L^{-1} (sea water, Black Sea) [33]
2,4-dinitroaniline	0.35 – 0.37 µg L^{-1} (reservoir and lake water, China) [29]
2,5-dichloroaniline	25.23-76.18 ng L^{-1} (river water, Turkey) [33]<br>6.55 – 8.14 ng L^{-1} (sea water, Black Sea) [33]
2,6-diethylaniline	0.37 – 7.28 ng L^{-1} (sea water, Black Sea) [33]
2-chloroaniline	0.52 ± 0.01 µg L^{-1} – 0.59 ± 0.01 µg L^{-1} (river water, Italy) [34]<br>0.15 – 0.18 µg L^{-1} (reservoir and lake water, China)[29]<br>95.88-192.95 ng L^{-1} (river water, Turkey) [33]
2-ethylaniline	0.53 – 15.97 ng L^{-1} (sea water, Black Sea) [33]
2-naphthylamine	57.64-66.54 ng L^{-1} (river water, Turkey) [33]<br>5.98 – 6.93 ng L^{-1} (sea water, Black Sea) [33]
2-nitroaniline	4.08-7.46 ng L^{-1} (river water, Turkey) [33]<br>1.23 – 2.31 ng L^{-1} (sea water, Black Sea) [33]<br>0.34 µg L^{-1} (lake water, China)[29]
3,4-dichloroaniline	1.5 - 3.4 ng L^{-1} (DWTP, Spain) [35]<br>1.13-1.94 ng L^{-1} (river water, Turkey) [33]<br>2.53 – 34.27 ng L^{-1} (sea water, Black Sea) [33]<br>0.097 µg L^{-1} -(lake water, China) [29]
3,5-dichloroaniline	2.72-12.66 ng L^{-1} (river water, Turkey) [33]<br>1.12 – 10.40 ng L^{-1} (sea water, Black Sea) [33]
3-aminophenol	3.09 – 6.55 ng L^{-1} (sea water, Black Sea) [33]
3-chloroaniline	1.3 - 2.7 ng L^{-1} – DWTP, Spain [35]<br>0.45 ± 0.08 µg L^{-1} (river water, Italy) [34]<br>4.64 – 8.86 ng L^{-1} (sea water, Black Sea)
3-nitroaniline	13.37-13.38 ng L^{-1} (river water, Turkey) [33]<br>2.78 – 9.18 ng L^{-1} (sea water, Black Sea) [33]
4-aminobiphenyl	2.46-4.84 ng L^{-1} (river water, Turkey) [33]<br>1.14 – 1.48 ng L^{-1} (sea water, Black Sea) [33]
4-aminophenol	1.81 – 4.28 ng L^{-1} (sea water, Black Sea) [33]
4-bromoaniline	2.22 – 18.85 ng L^{-1} (sea water, Black Sea) [33]
4-chloroaniline	2.0 ± 0.2 µg L-1 (river water, Italy) [34]<br>0.66-0.82 ng L^{-1} (river water, Turkey) [33]
4-ethylaniline	19.38-20.52 ng L^{-1} (river water, Turkey) [33]<br>0.46 – 1.04 ng L^{-1} (sea water, Black Sea) [33]
4-Methyl-o-phenylenediamine	3.88 – 3.98 ng L^{-1} (sea water, Black Sea) [33]
aniline	2.2 – 12 ng L^{-1} – (DWTP), Spain) [35]

	0.03 ng mL^{-1} (river water, Iran)[32]<br>2.48 – 23.78 ng L^{-1} (sea water, Black Sea) [33]<br>4.5 µg L^{-1} (river water, India) [36]
benzylamine	26.59 – 42.74 ng L^{-1} (sea water, Black Sea) [33]
diphenylamine	80.22-102.99 ng L^{-1} (river water, Turkey) [33]<br>0.13 – 3.05 ng L^{-1} (sea water, Black Sea) [33]
IPPD	<LOD-8.9 ng L^{-1} (river water, China) [37]<br>0.07* ng/L (surface water, China)[38]
m-Toluidine	1.25-1.89 ng L-1 (river water, Turkey) [33]<br>0.38 – 1.46 ng L^{-1} (sea water, Black Sea) [33]
N-Methylaniline	0.49 – 54.15 ng L^{-1} (sea water, Black Sea) [33]
o-toluidine	<10 µg L^{-1} (river water, India) [36]<br>>100 ng/L (Elbe River, Germany)[40]
phenylethylamine	3.26 – 20.87 ng L^{-1} (sea water, Black Sea) [33]
6PPD	<4.0-72 ng L^{-1} (river water, China) [37]<br>0.31-1.07 ng L^{-1} (Zhujiang River, China) [39]<br>0.27-1.29 ng L^{-1} (Dongjiang River, China) [39]
o-anisidine	>100 ng L^{-1} (Elbe River, Germany)[40]
4-isopropylaniline	500 ng L^{-1} (Ebro River, Spain)[42]
MeIQx	ND-365 Blue Rayon equivalents (Yodo River, Japan)[50]

*median

References

(1) Muz, M.; Ost, N.; Kühne, R.; Schüürmann, G.; Brack, W.; Krauss, M. Nontargeted Detection and Identification of (Aromatic) Amines in Environmental Samples Based on Diagnostic Derivatization and LC-High Resolution Mass Spectrometry. *Chemosphere* **2017**, *166*, 300–310. https://doi.org/10.1016/j.chemosphere.2016.09.138.

(2) Jin, R.; Venier, M.; Chen, Q.; Yang, J.; Liu, M.; Wu, Y. Amino Antioxidants: A Review of Their Environmental Behavior, Human Exposure, and Aquatic Toxicity. *Chemosphere* **2023**, *317*, 137913. https://doi.org/10.1016/J.CHEMOSPHERE.2023.137913.

(3) Zoroufchi Benis, K.; Behnami, A.; Minaei, S.; Brinkmann, M.; N. McPhedran, K.; Soltan, J. Environmental Occurrence and Toxicity of 6PPD Quinone, an Emerging Tire Rubber-Derived Chemical: A Review. *Environmental Science & Technology Letters* **2023**, *10* (10), 815–823. https://doi.org/10.1021/acs.estlett.3c00521.

(4) Hua, X.; Wang, D. Tire-Rubber Related Pollutant 6-PPD Quinone: A Review of Its Transformation, Environmental Distribution, Bioavailability, and Toxicity. *J Hazard Mater* **2023**, *459*, 132265. https://doi.org/10.1016/J.JHAZMAT.2023.132265.

(5) Ward, E. M.; Sabbioni, G.; Debord, D. G.; Teass, A. W.; Brown, K. K.; Talaska, G. G.; Roberts, D. R.; Ruder, A. M.; Streicher, R. P. *Monitoring of Aromatic Amine Exposures in Workers at a Chemical Plant With a Known Bladder Cancer Excess*; 1991. https://academic.oup.com/jnci/article/88/15/1046/892703.

(6) Zhu, J.; Aikawa, B. Determination of Aniline and Related Mono-Aromatic Amines in Indoor Air in Selected Canadian Residences by a Modified Thermal Desorption GC/MS Method. *Environ Int* **2004**, *30* (2), 135–143. https://doi.org/10.1016/S0160-4120(03)00168-5.

(7) Akyüz, M. Simultaneous Determination of Aliphatic and Aromatic Amines in Indoor and Outdoor Air Samples by Gas Chromatography–Mass Spectrometry. *Talanta* **2007**, *71* (1), 486–492. https://doi.org/10.1016/J.TALANTA.2006.10.028.

(8) To, W. M.; Lau, Y. K.; Yeung, L. L. Emission of Carcinogenic Components from Commercial Kitchens in Hong Kong. *Indoor and Built Environment* **2007**, *16* (1), 29–37. https://doi.org/10.1177/1420326X06074500.

(9) Palmiotto, G.; Pieraccini, G.; Moneti, G.; Dolara, P. Determination of the Levels of Aromatic Amines in Indoor and Outdoor Air in Italy. *Chemosphere* **2001**, *43* (3), 355–361. https://doi.org/10.1016/S0045-6535(00)00109-0.

(10) Lucaire, V.; Schwartz, J. J.; Delhomme, O.; Ocampo-Torres, R.; Millet, M. A Sensitive Method Using SPME Pre-Concentration for the Quantification of Aromatic Amines in Indoor Air. *Anal Bioanal Chem* **2018**, *410* (7), 1955–1963. https://doi.org/10.1007/s00216-018-0862-8.

(11) Chinthakindi, S.; Kannan, K. Primary Aromatic Amines in Indoor Dust from 10 Countries and Associated Human Exposure. *Environ Int* **2021**, *157*, 106840. https://doi.org/10.1016/J.ENVINT.2021.106840.

(12) Liu, R.; Li, Y.; Lin, Y.; Ruan, T.; Jiang, G. Emerging Aromatic Secondary Amine Contaminants and Related Derivatives in Various Dust Matrices in China. *Ecotoxicol Environ Saf* **2019**, *170*, 657–663. https://doi.org/10.1016/J.ECOENV.2018.12.036.

(13) Huang, W.; Shi, Y.; Huang, J.; Deng, C.; Tang, S.; Liu, X.; Chen, D. Occurrence of Substituted P-Phenylenediamine Antioxidants in Dusts. *Environmental Science & Technology Letters* **2021**, *8* (5), 381–385. https://doi.org/10.1021/acs.estlett.1c00148.

(14) Wu, Y.; Venier, M.; A. Hites, R. Broad Exposure of the North American Environment to Phenolic and Amino Antioxidants and to Ultraviolet Filters. *Environmental Science & Technology* **2020**, *54* (15), 9345–9355. https://doi.org/10.1021/acs.est.0c04114.

(15) Tan, H.; Yang, L.; Huang, Y.; Tao, L.; Chen, D. "Novel" Synthetic Antioxidants in House Dust from Multiple Locations in the Asia-Pacific Region and the United States. *Environmental Science & Technology* **2021**, *55* (13), 8675–8682. https://doi.org/10.1021/acs.est.1c00195.

(16) Guo, Z.; Cheng, Z.; Zhang, S.; Zhu, H.; Zhao, L.; Baqar, M.; Wang, L.; Sun, H. Unexpected Exposure Risks to Emerging Aromatic Amine Antioxidants and P-Phenylenediamine Quinones to Residents: Evidence from External and Internal Exposure as Well as Hepatotoxicity Evaluation. *Environment & Health* **2024**, *0* (0). https://doi.org/10.1021/envhealth.3c00205.

(17) Liang, B.; Li, J.; Du, B.; Pan, Z.; Liu, L.-Y.; Zeng, L. E-Waste Recycling Emits Large Quantities of Emerging Aromatic Amines and Organophosphites: A Poorly Recognized Source for Another Two Classes of Synthetic Antioxidants. *Environmental Science & Technology Letters* **2022**, *9* (7), 625–631. https://doi.org/10.1021/acs.estlett.2c00366.

(18) Zhang, Y.; Xu, C.; Zhang, W.; Qi, Z.; Song, Y.; Zhu, L.; Dong, C.; Chen, J.; Cai, Z. P-Phenylenediamine Antioxidants in PM2.5: The Underestimated Urban Air Pollutants. *Environmental Science & Technology* **2021**, *56* (11), 6914–6921. https://doi.org/10.1021/acs.est.1c04500.

(19) Cao, G.; Wang, W.; Zhang, J.; Wu, P.; Zhao, X.; Yang, Z.; Hu, D.; Cai, Z. New Evidence of Rubber-Derived Quinones in Water, Air, and Soil. *Environmental Science & Technology* **2022**, *56* (7), 4142–4150. https://doi.org/10.1021/acs.est.1c07376.

(20) Wang, W.; Cao, G.; Zhang, J.; Wu, P.; Chen, Y.; Chen, Z.; Qi, Z.; Li, R.; Dong, C.; Cai, Z. Beyond Substituted P-Phenylenediamine Antioxidants: Prevalence of Their Quinone Derivatives in PM2.5. *Environmental Science & Technology* **2022**, *56* (15), 10629–10637. https://doi.org/10.1021/acs.est.2c02463.

(21) Deng, C.; Huang, J.; Qi, Y.; Chen, D.; Huang, W. Distribution Patterns of Rubber Tire-Related Chemicals with Particle Size in Road and Indoor Parking Lot Dust. *Science of The Total Environment* **2022**, *844*, 157144. https://doi.org/10.1016/J.SCITOTENV.2022.157144.

(22) Klöckner, P.; Seiwert, B.; Weyrauch, S.; Escher, B. I.; Reemtsma, T.; Wagner, S. Comprehensive Characterization of Tire and Road Wear Particles in Highway Tunnel Road Dust by Use of Size and Density Fractionation. *Chemosphere* **2021**, *279*, 130530. https://doi.org/10.1016/J.CHEMOSPHERE.2021.130530.

(23) Hiki, K.; Yamamoto, H. Concentration and Leachability of N-(1,3-Dimethylbutyl)-N′-Phenyl-p-Phenylenediamine (6PPD) and Its Quinone Transformation Product (6PPD-Q) in Road Dust Collected in Tokyo, Japan. *Environmental Pollution* **2022**, *302*, 119082. https://doi.org/10.1016/J.ENVPOL.2022.119082.

(24) Onuska, F. I. ; T. K. A. ; M. R. J. Analysis of Aromatic Amines in Industrial Wastewater by Capillary Gas Chromatography-Mass Spectrometry. *Water Quality Research Journal* **2000**, *35* (2), 245–262.

(25) Li, R.; Zhang, Y.; Lee, C. C.; Lu, R.; Huang, Y. Development and Validation of a Hydrophilic Interaction Liquid Chromatographic Method for Determination of Aromatic Amines in Environmental Water. *J Chromatogr A* **2010**, *1217* (11), 1799–1805. https://doi.org/10.1016/J.CHROMA.2010.01.049.

(26) Muz, M.; Paul Dann, J.; Jäger, F.; Brack, W.; Krauss, M. Identification of Mutagenic Aromatic Amines in River Samples with Industrial Wastewater Impact. *Environmental Science & Technology* **2017**, *51* (8), 4681–4688. https://doi.org/10.1021/acs.est.7b00426.

(27) Albahnasawi, A.; Yüksel, E.; Gürbulak, E.; Duyum, F. Fate of Aromatic Amines through Decolorization of Real Textile Wastewater under Anoxic-Aerobic Membrane Bioreactor. *J Environ Chem Eng* **2020**, *8* (5), 104226. https://doi.org/10.1016/J.JECE.2020.104226.

(28) Vacchi, F. I.; Vendemiatti, J. A. S.; Brosselin, V.; Ferreira da Silva, B.; Maria, M. V.; DeMeo, M.; Bony, S.; Devaux, A.; Umbuzeiro, G. A. Combining Different Assays and Chemical Analysis to Characterize the Genotoxicity of Waters Impacted by Textile Discharges. *Environ Mol Mutagen* **2016**, *57* (7), 559–571. https://doi.org/10.1002/em.22034.

(29) Wu, J.; Huang, Y.; Huang, X. Efficient Trap of Polar Aromatic Amines in Environmental Waters by Electroenhanced Solid Phase Microextraction Based on Porous Monolith Doped with Carboxylic Carbon Nanotubes. *Sep Purif Technol* **2022**, *282*, 120067. https://doi.org/10.1016/J.SEPPUR.2021.120067.

(30) Basheer, C. Liquid Chromatographic Determination of Aromatic Amines in Water Samples after Gold Nanoparticles Coated Membrane Microextraction. *Asian Journal of Chemistry* **2020**, *32* (9), 2389–2396. https://doi.org/10.14233/ajchem.2020.22292.

(31) Cao, G.; Wang, W.; Zhang, J.; Wu, P.; Qiao, H.; Li, H.; Huang, G.; Yang, Z.; Cai, Z. Occurrence and Fate of Substituted P-Phenylenediamine-Derived Quinones in Hong Kong Wastewater Treatment Plants. *Environmental Science & Technology* **2023**, *57* (41), 15635–15643. https://doi.org/10.1021/acs.est.3c03758.

(32) Amiri, A.; Saadati-Moshtaghin, H. R.; Zonoz, F. M.; Targhoo, A. Preparation and Characterization of Magnetic Wells–Dawson Heteropoly Acid Nanoparticles for Magnetic Solid-Phase Extraction of Aromatic Amines in Water Samples. *J Chromatogr A* **2017**, *1483*, 64–70. https://doi.org/10.1016/J.CHROMA.2016.12.083.

(33) Akyüz, M.; Ata, Ş. Simultaneous Determination of Aliphatic and Aromatic Amines in Water and Sediment Samples by Ion-Pair Extraction and Gas Chromatography–Mass

Spectrometry. *J Chromatogr A* **2006**, *1129* (1), 88–94. https://doi.org/10.1016/J.CHROMA.2006.06.075.

(34) Gosetti, F.; Chiuminatto, U.; Zampieri, D.; Mazzucco, E.; Marengo, E.; Gennaro, M. C. A New On-Line Solid Phase Extraction High Performance Liquid Chromatography Tandem Mass Spectrometry Method to Study the Sun Light Photodegradation of Mono-Chloroanilines in River Water. *J Chromatogr A* **2010**, *1217* (20), 3427–3434. https://doi.org/10.1016/J.CHROMA.2010.02.080.

(35) Jurado-Sánchez, B.; Ballesteros, E.; Gallego, M. Occurrence of Aromatic Amines and N-Nitrosamines in the Different Steps of a Drinking Water Treatment Plant. *Water Res* **2012**, *46* (14), 4543–4555. https://doi.org/10.1016/j.watres.2012.05.039.

(36) Jain, A.; Reddy-Noone, K.; Pillai, A. K. K. V.; Verma, K. K. Conversion to Isothiocyanates via Dithiocarbamates for the Determination of Aromatic Primary Amines by Headspace-Solid Phase Microextraction and Gas Chromatography. *Anal Chim Acta* **2013**, *801*, 48–58. https://doi.org/10.1016/J.ACA.2013.09.046.

(37) Zhu, J.; Guo, R.; Ren, F.; Jiang, S.; Jin, H. Occurrence and Partitioning of P-Phenylenediamine Antioxidants and Their Quinone Derivatives in Water and Sediment. *Science of The Total Environment* **2024**, *914*, 170046. https://doi.org/10.1016/J.SCITOTENV.2024.170046.

(38) Zhang, R.; Zhao, S.; Liu, X.; Tian, L.; Mo, Y.; Yi, X.; Liu, S.; Liu, J.; Li, J.; Zhang, G. Aquatic Environmental Fates and Risks of Benzotriazoles, Benzothiazoles, and p-Phenylenediamines in a Catchment Providing Water to a Megacity of China. *Environ Res* **2023**, *216*, 114721. https://doi.org/10.1016/J.ENVRES.2022.114721.

(39) Zhang, H. Y.; Huang, Z.; Liu, Y. H.; Hu, L. X.; He, L. Y.; Liu, Y. S.; Zhao, J. L.; Ying, G. G. Occurrence and Risks of 23 Tire Additives and Their Transformation Products in an Urban Water System. *Environ Int* **2023**, *171*, 107715. https://doi.org/10.1016/J.ENVINT.2022.107715.

(40) Reifferscheid, G.; Grummt, T. Genotoxicity in German Surface Waters - Results of a Collaborative Study. Water, Air, and Soil Pollution 2000, 123 (1), 67–79. https://doi.org/10.1023/A:1005201207088.

(41) Barreto, P.; Lemes, M.; Jimenez, J.; Mack, E. E.; Henderson, J.; Freedman, D. L. Evaluation of Strategies to Remediate Mixed Wastes at an Industrial Site in Brazil. Groundwater Monitoring & Remediation 2023, 43 (3), 93–107. https://doi.org/10.1111/gwmr.12607.

(42) Ormad, M. P.; Miguel, N.; Claver, A.; Matesanz, J. M.; Ovelleiro, J. L. Pesticides Removal in the Process of Drinking Water Production. Chemosphere 2008, 71 (1), 97–106. https://doi.org/10.1016/j.chemosphere.2007.10.006.

(48) Liu, R.; Mabury, S. A. Identification of Photoinitiators, Including Novel Phosphine Oxides, and Their Transformation Products in Food Packaging Materials and Indoor Dust in Canada. Environ. Sci. Technol. 2019, 53 (8), 4109–4118. https://doi.org/10.1021/acs.est.9b00045.

(49) Liu, R.; Lin, Y.; Hu, F.; Liu, R.; Ruan, T.; Jiang, G. Observation of Emerging Photoinitiator Additives in Household Environment and Sewage Sludge in China. Environ. Sci. Technol. 2016, 50 (1), 97–104. https://doi.org/10.1021/acs.est.5b04977.

(50) Ohe, T. Quantification of Mutagenic/Carcinogenic Heterocyclic Amines, MeIQx, Trp-P-1, Trp-P-2 and PhIP, Contributing Highly to Genotoxicity of River Water. Mutation Research/Genetic Toxicology and Environmental Mutagenesis 1997, 393 (1), 73–79. https://doi.org/10.1016/S1383-5718(97)00087-9.
